# Light-induced morphological alteration in anthocyanin-accumulating vacuoles of maize cells

**DOI:** 10.1186/1471-2229-5-7

**Published:** 2005-05-20

**Authors:** Niloufer G Irani, Erich Grotewold

**Affiliations:** 1 Department of Plant Cellular and Molecular Biology and Plant Biotechnology Center, The Ohio State University, Columbus, OH 43210, USA

## Abstract

**Background:**

Plant pigmentation is affected by a variety of factors. Light, an important plant developmental signal, influences the accumulation of anthocyanins primarily through the activation of the transcription factors that regulate the flavonoid biosynthetic pathway. In this study, we utilized maize Black Mexican Sweet (BMS) cells expressing the R and C1 regulators of anthocyanin biosynthesis from a light-insensitive promoter as a means to investigate the existence of additional levels of control of pigmentation by light.

**Results:**

BMS cells expressing the R and C1 regulators from the *CaMV 35S *constitutive promoter accumulate anthocyanins when grown in complete darkness, suggesting that the transcription factors R and C1 are sufficient for the transcription of the genes corresponding to the structural enzymes of the pathway, with no requirement for additional light-induced regulators. Interestingly, light induces a "darkening" in the color of the purple anthocyanin pigmentation of transgenic BMS cells expressing R and C1. This change in the pigment hue is not associated with a variation in the levels or types of anthocyanins present, or with an alteration of the transcript levels of several flavonoid biosynthetic genes. However, cytological observations show that light drives unexpected changes in the morphology and distribution of the anthocyanins-containing vacuolar compartments.

**Conclusion:**

By uncoupling the effect of light on anthocyanin accumulation, we have found light to induce the fusion of anthocyanin-containing vacuoles, the coalescence of anthocyanic vacuolar inclusion (AVI)-like structures contained, and the spread of anthocyanins from the inclusions into the vacuolar sap. Similar light-induced alterations in vacuolar morphology are also evident in the epidermal cells of maize floral whorls accumulating anthocyanins. Our findings suggest a novel mechanism for the action of light on the vacuolar storage of anthocyanin.

## Background

Anthocyanins, the coloured end product of the flavonoid pathway, play an important role in attracting insects or animals for pollination and seed dispersal. In addition, they play roles as anti-oxidants and in protecting DNA and the photosynthetic apparatus from high radiation fluxes [1]. Other possible functions of anthocyanins, such as the protection against cold stress or providing drought resistance, are likely to be associated with activities restricted to particular classes of plants [2].

The biosynthesis of the flavonoids, a large phenylpropanoid-derived group of phenolic compounds, provides one of the best described plant metabolic pathways, with many of the structural and regulatory genes in the pathway identified and cloned [3,4]. Less is known regarding the mechanisms by which the water-soluble anthocyanins are transported from their site of synthesis, the cytoplasmic surface of the endoplasmic reticulum [5,6], to the vacuole, where they are usually sequestered [7]. Plant vacuoles are highly dynamic, multifunctional organelles that are the primary storage and turnover sites of macromolecules. These membrane-bound organelles, which can occupy up to 90% of the total cellular volume, are integral part of the endomembrane system, serving as the terminal products of the secretory pathway [8].

Several plant species store anthocyanins within vacuolar inclusions that have been loosely termed anthocyanoplasts which have been observed to start as vesicles in the cytoplasm and appear to be membrane bound [9,10]. More recently, the intravacuolar structures observed in the flower petals of various plants, including carnation and lisianthus, were termed anthocyanic vacuolar inclusions, or AVIs [11]. These inclusions were suggested to be membrane-less, proteinaceous matrixes that acted as anthocyanin traps, preferentially for anthocyanidin 3, 5-diglycosides [11] or acylated anthocyanins [12]. Once in the vacuole, many factors influence the *in vivo *pigmentation provided by anthocyanins, with important consequences for the eco-physiology of plants [13,14]. Some of these factors include the particular type of anthocyanidin present (for e.g., pelargonidin cyanidin, or myricetin), the shape of the cells in which the pigments accumulate [15], the concentration of the pigment, the formation of complexes between anthocyanins and co-pigments [16], and the vacuolar pH[17,18].

Environmental conditions are known to induce the accumulation of anthocyanin pigments across the major groups of higher plants, of them light being the best studied [2,19,20]. In *Arabidopsis*, the anthocyanin pathway is regulated in a circadian fashion, with flavonoid genes peaking at the end of the dark cycle, likely preparing plants for daybreak [21]. In maize, members of the PL1/C1 R2R3 MYB and B/R bHLH families of regulatory proteins cooperate for the regulation of anthocyanin pigments [3] and are necessary for the expression of the anthocyanin biosynthetic genes *c2 *(chalcone synthase), *chi1 *(chalcone isomerase), *f3h *(flavanone 3-hydroxylase) *a1 *(dihydroflavonol 4-reductase), *a2 *(leucoanthocyanidin dioxygenase/anthocyanin synthase), *bz1 *(UDP glucose:flavonoid 3-*O*-glucosyl transferase) and *bz2 *(glutathione *S*-transferase) [19]. The light-induced expression of members of these R2R3 MYB and bHLH classes of transcription factors has been proposed to be responsible for the induction of anthocyanins in maize by light [22-26].

Here, we investigated whether light affects the accumulation of maize anthocyanin pigmentation at a level other than through the activation of the known R2R3 MYB and bHLH regulators of the pathway. Towards this goal, we analyzed previously described [27] transgenic Black Mexican Sweet (BMS) maize cells in culture expressing the *Zea mays *R and C1 regulators from a constitutive, light insensitive *CaMV 35S *promoter (BMS^35S::R+35S::C1^). We show here that BMS^35S::R+35S::C1 ^cells are red, even when grown in complete darkness. Upon light treatment, there is a darkening of the color of the BMS^35S::R+35S::C1 ^cells, without an appreciable increase in the quantity of anthocyanins or in the type of anthocyanidins present. Consistent with these findings, the steady-state levels of several anthocyanin biosynthetic genes does not increase upon light treatment. Interestingly, at the subcellular level, light induces an alteration in the way the anthocyanins are distributed within vacuolar compartments. A similar alteration in the morphology of anthocyanin-accumulating vacuoles is observed when maize tassel glumes are irradiated with light, suggesting that the phenomena observed in BMS^35S::R+35S::C1 ^cells in culture also occurs *in planta*. Together, our findings suggest a novel mechanism for the action of light on the packaging of anthocyanins in the vacuole and in subvacuolar compartments. This effect of light could only be uncovered after making the biosynthesis of anthocyanins independent of light.

## Results

### BMS cells expressing the transcription factors R and C1 accumulate anthocyanins in the dark

**Figure 1:**
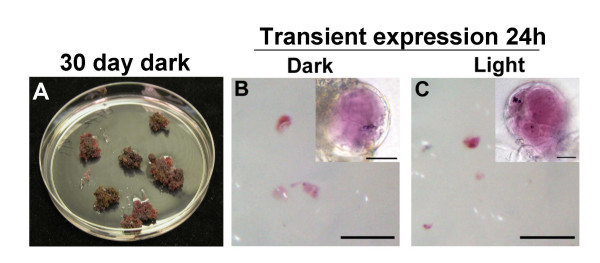
**Anthocyanins accumulate in maize BMS^35S::R+35S::C1 ^cells in the dark. **(A) Dark-grown BMS^35S::R+35S::C1 ^cells expressing the R and C1 anthocyanin regulators from the *CaMV 35S *promoter accumulate anthocyanins. Transient expression of the R and C1 regulators in BMS cells by microprojectile bombardment induce anthocyanins in the dark (B) or the light (C). The magnification bars represent 200 μm. The bar in the inset DIC image is 20 μm.

To determine whether the light control of the maize anthocyanin pathway is mediated by the expression of the B/R and C1/PL regulators and/or the biosynthetic genes [23], we investigated the pigmentation of BMS cells expressing the R and C1 genes from the constitutive *CaMV 35S *promoter (BMS^35S::R+35S::C1^) [27]. BMS^35S::R+35S::C1 ^cells grown in complete darkness for 30 days were fully pigmented with anthocyanins (Fig. 1A). The bombardment of BMS cells with the R and C1 regulators driven from the 35S promoter (p35SR + p35SC1) resulted in the accumulation of red cells within 15 hours, even when cells were kept in complete darkness after bombardment (compare Fig. 1B and 1C). These results indicate that the constitutive expression of the R and C1 regulators is sufficient for the activation of the pathway, even in the absence of light.

### BMS^35S::R+35S::C1 ^cells darken in the light

**Figure 2:**
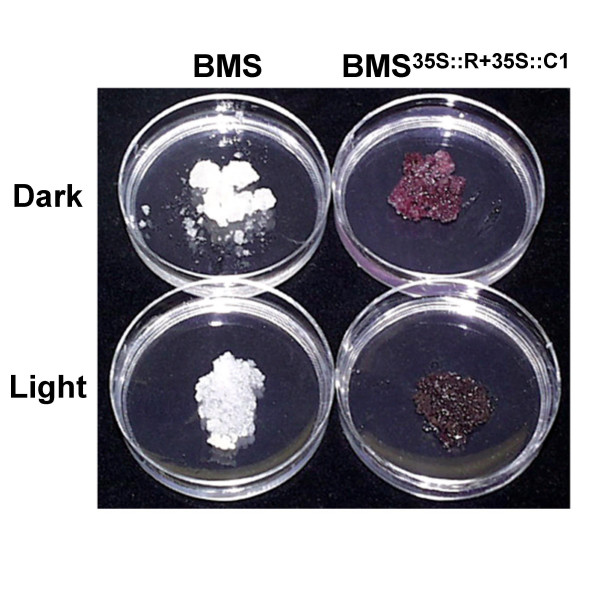
**Light induces the darkening of anthocyanin pigmentation. **Images of BMS control and BMS^35S::R+35S::C1 ^cells grown for ten days under total darkness (Dark) or light (Light) conditions.

To investigate whether light has any additional effect on the pigmentation present in BMS^35S::R+35S::C1 ^cells, we compared the color of BMS and BMS^35S::R+35S::C1 ^cells grown for six days in complete darkness or under light conditions (see Methods). BMS^35S::R+35S::C1 ^cells grown in the light showed a visual darkening, when compared to cells grown in the dark (Fig. 2, BMS^35S::R+35S::C1^). However, light did not induce any visible difference in the white-yellow color of the control BMS cells not expressing the C1 and R regulators (Fig. 2, BMS). We quantified these visual color differences *in vivo *using a reflectometer and the CIELAB color space value system. L* values, representing the lightness level, were reproducibly not affected by light (Table 1). The a* values for BMS^35S::R+35S::C1 ^cells were positive (+a), consistent with the red color characteristic of these cells. There was however a significant (p < 0.05) reduction in the a-values when the BMS^35S::R+35S::C1 ^cells were exposed to light, which was observed in each of the three times that the experiment was performed (Table 1). Although the b* values, contributing to yellow (+b) or blue (-b), were significantly different (p < 0.05) between the dark and light grown BMS^35S::R+35S::C1 ^cells, they hovered near the zero value, suggesting a low contribution to the overall observed color shift. Thus, the a* and the b* values observed corresponded to a quantifiably red color (dull red), with a decreased degree of redness in the light, which is in agreement with our visual observations (Fig. 2). In the absence of a change in the L* value, the apparent darkening of the cells is likely caused by the spectral shift of the reflected light towards the less red.

**Table 1:** Reflectance analysis and L* a* b* values of dark- and light-grown BMS^35S::R+35S::C1 ^cells in the CIELAB color scale. The a* value contributes to red (a+) or green (a-), the b* value contributes to yellow (+b) or blue (-b) and L* represent the lightness level.

**BMS^35S::R+35S::C1^**	L*	a*	b*

**Dark**	21.42 ± 1.29	12.06 ± 1.51	0.45 ± 0.48
**Light**	21.01 ± 0.93	7.02 ± 0.88	-0.45 ± 0.21

### The anthocyanin contents or mRNA steady state levels of biosynthetic genes are not altered by white light in BMS^35S::R+35S::C1 ^cells

**Figure 3:**
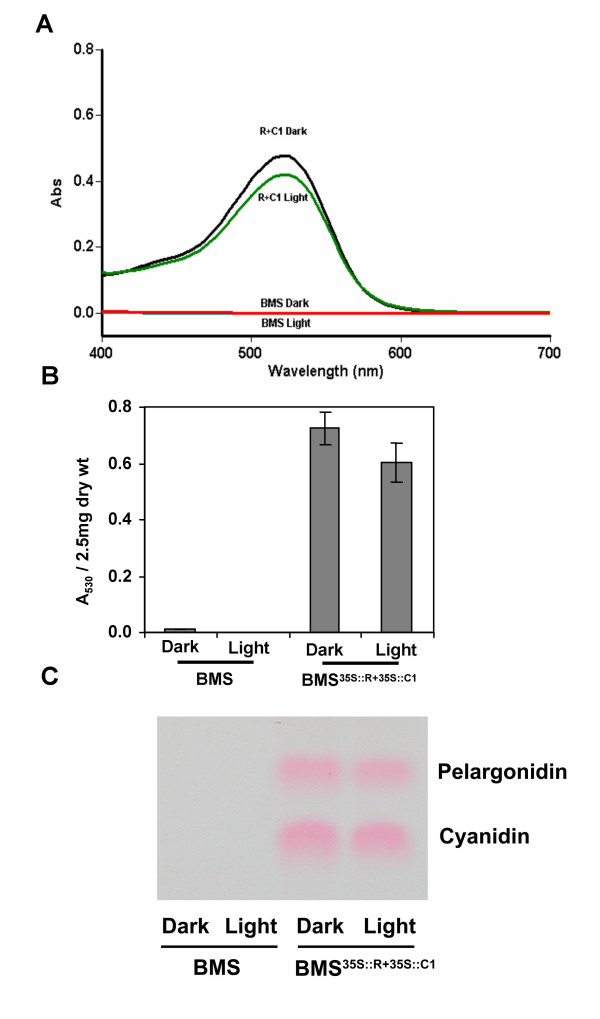
**Similar quantities of cyaniding and pelargonidin accumulate in dark and light-grown BMS^35S::R+35S::C1 ^cells. **(A) Spectral profile of methanol-HCL extracts of dark- (black line) or light-grown (green line) BMS^35S::R+35S::C1 ^cells. The red line corresponds to the spectra of control BMS extracts. (B) Quantitation of anthocyanins in dark- and light-grown BMS^35S::R+35S::C1 ^cells. Bars represent the SD of measurements obtained in three independent experiments. (C) Qualitative analysis of the anthocyanins present in dark- and light-grown BMS^35S::R+35S::C1 ^cells by TLC.

To investigate whether the change in color observed between dark- and light grown BMS^35S::R+35S::C1 ^cells is due to an alteration in the quality or quantity of the pigments, anthocyanins were extracted and quantified. Methanolic extracts of the cells, normalized for dry weight, were analyzed spectrophotometrically. The absorption spectra of pigments obtained from acidic methanol extracts of dark- and light-treated cells showed identical profiles and very similar absorbance values at 530 nm (Fig. 3A, B). To determine whether light induced an alteration in the type of anthocyanidin present, total anthocyanidins were extracted and separated by thin layer chromatography (TLC). Similar levels of cyanidin and pelargonidin are present in light- and dark treated BMS^35S::R+35S::C1 ^cells (Fig. 3C), consistent with the two major types of anthocyanidins previously described in BMS^35S::R+35S::C1 ^cells [28].

**Figure 4:**
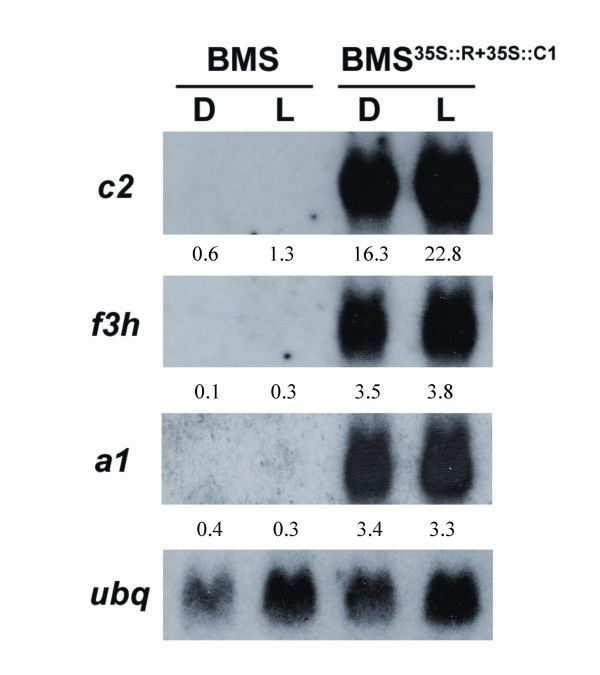
**Northern analysis of flavonoid biosynthetic genes show no alterations in the steady-state mRNA levels induced by light in BMS^35S::R+35S::C1 ^cells. **Total RNA from dark- (D) and light-grown (L) control BMS and BMS^35S::R+35S::C1 ^cells were analyzed by Northern with probes corresponding to the *c2 *(chalcone synthase), *f3h *(flavanone 3-hydroxylase) and *a1 *(dihyroflavonol 4-reductase) genes. Ubiquitin (*ubq*) was used as a normalizing control. The numbers indicate the relative hybridization signal (in arbitrary units) obtained with each probe, normalized for the corresponding signal obtained with *ubq*.

In BMS cells, R and C1 are known to activate several flavonoid biosynthetic genes [27]. To investigate whether the activation of these genes was further enhanced by the light treatment, total RNA was extracted from BMS^35S::R+35S::C1 ^cells incubated for six days in the light or the dark and analyzed by RNA gel blots (Northern blots, Fig. 4). No significant alteration in the steady-state mRNA levels for chalcone synthase (*c*2, Fig. 4), flavanone 3-hydroxylase (*f3h*, Fig. 4) or dihydroflavonol 4-reductase (*a1*, Fig. 4) was observed for the light grown BMS^35S::R+35S::C1 ^cells. The control BMS cells showed no mRNA accumulation for these genes (Fig. 4). Together, these results suggest that the darkening of the BMS^35S::R+35S::C1 ^cells upon light treatment is not due to an alteration in the quantity or quality of the anthocyanin pigments present.

### White light induces alterations in the sub-cellular distribution and vacuolar organization of anthocyanins in BMS^35S::R+35S::C1 ^cells

**Figure 5:**
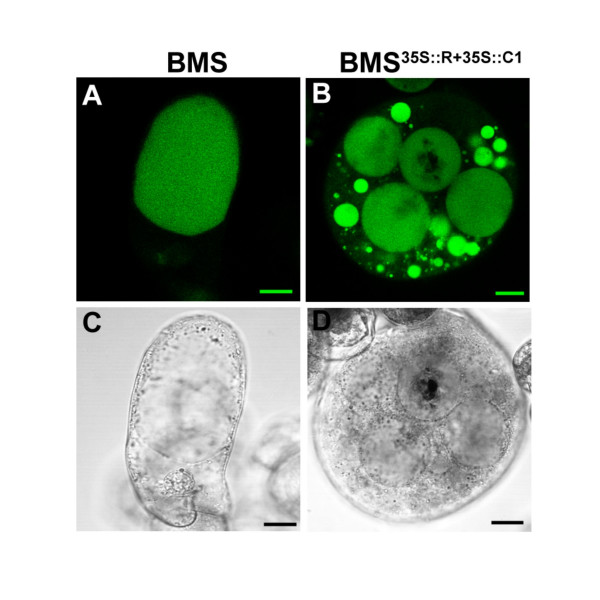
**BMS^35S::R+35S::C1 ^cells have multiple vacuoles. **Laser scanning confocal microscopy (false colored) images of (A) BMS and (B) BMS^35S::R+35S::C1 ^cells loaded with 10 μM of the vacuolar dye BCECF-AM. Laser scanning confocal 'light transmitted' images of (C) BMS and (D) BMS^35S::R+35S::C1 ^cells are shown in black and white. The bar represent 20 μm.

Alterations in rose flower pigmentation were associated previously with the formation of AVI-like structures [29]. To investigate whether a similar alteration in the packing of anthocyanins could be with the light-induced hue alteration of BMS^35S::R+35S::C1 ^cells, we investigated the sub-cellular morphology of dark- and light-treated BMS^35S::R+35S::C1 ^cells. To unequivocally visualize the vacuole(s), BMS and BMS^35S::R+35::C1 ^were stained with the cell permeable, acetoxymethyl derivative of the fluorescent vacuolar dye, 2',7'-bis(2-carboxyethyl)-5(6)-carboxyfluorescein (BCECF-AM). In the control BMS cells, there are typically one to a few large vacuolar compartments (Fig. 5A, C). In contrast, BMS^35S::R+35::C1 ^cells are always multi-vacuolated (Fig. 5B, D). Unfortunately, anthocyanins concentrated in the vacuolar inclusions quench the fluorescence of the BCECF-AM dye, as observed in some of the larger vacuoles (Fig. 5B).

**Figure 6:**
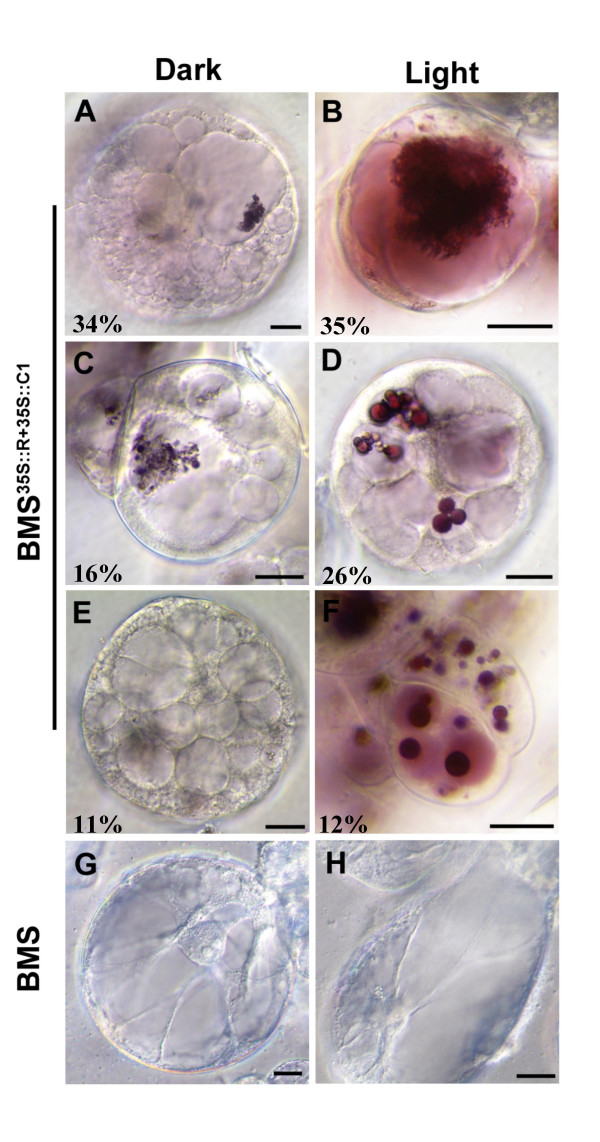
**Light induces alterations in the distribution of anthocyanins within vacuolar compartments. **Representative DIC images of six day old (A, C, E) dark-grown and (B, D, F) light-grown BMS^35S::R+35S::C1 ^cells. BMS cells grown in the (G) dark or (H) light show no significant alterations. Numbers correspond to the percentage values indicated in Table 2. The bar represents 20 μm.

Within the vacuole, anthocyanins accumulate in inclusions that, when observed under polarized light, appear round and regular in shape or aggregated, like an intertwine of fine strings with blebs (Fig. 6). The number of vacuoles and inclusions per vacuole are dramatically affected by light in BMS^35S::R+35S::C1 ^cells. In dark-treated BMS^35S::R+35::C1 ^cells, the major representative cell type (Fig. 6A, representing 34% of all the 239 cells observed), had a range of 20 to 30 observable vacuoles (Table 2). In these cells, the anthocyanins were present mainly in the rounded vacuolar inclusions with a characteristic pale pink coloration of the vacuolar sap, a phenomenon called here "anthocyanin spread". The next two most abundant cell types present in dark-treated BMS^35S::R+35::C1 ^cells, corresponding to 16% and 11% of all the cells, had a range of 10–20 or 20–30 visually observable vacuoles per cell, respectively (Table 2). Cells of these groups are characterized by no observable anthocyanin spread in the vacuolar sap under the light microscope, yet had red or pale red anthocyanin inclusions in the vacuole (Fig. 6C, E).

**Table 2:** Vacuole distribution in dark- and light-grown BMS^35S::R+35S::C1 ^cells. The classification is based on the number of vacuoles and the presence or absence of anthocyanins in the vacuolar sap. The A-F letters correspond to the panels in Fig. 6, and the presence (+) or absence (-) of anthocyanins in the vacuolar sap (spread) is indicated.

		**% Representation**	**No. of vacuoles (range)**	**Anthocyanin spread**	
**35S::R+C1 Dark**					(n = 239)
	**A**	34%	20–30	+	
	**C**	16%	10–20	-	
	**E**	11%	20–30	-	

**35S::R+C1 Light**					(n = 400)
	**B**	35%	1–10	++	
	**D**	26%	10–20	+	
	**F**	12%	1–10	+++	

In the light, the majority of the BMS^35S::R+35::C1 ^cells (35%, Table 2) show one to ten pigmented vacuoles with red inclusions that appeared most of time diffuse and like "tangled strings" (Fig, 6B). These structures were determined to be 0.1 to 0.3 μm in diameter, appeared in certain cells to be branched with bleb-like structures at their ends. A significant population of cells (26%, Table 2) had 10–20 vacuoles, lightly colored with discrete deeply pigmented, spherical inclusions (Fig. 6D). The third most abundant class of BMS^35S::R+35::C1 ^cells present in the light (12%, Table 2) showed deeply pigmented vacuoles and enlarged inclusions (Fig. 6F).

**Figure 7:**
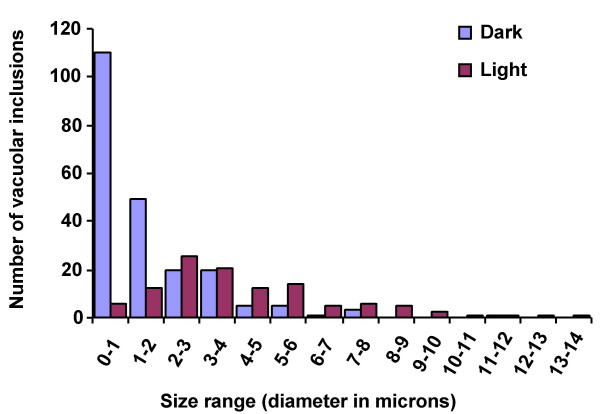
**Comparison of the size distribution of vacuolar inclusion containing anthocyanins in BMS^35S::R+35S::C1 ^cells grown in the light or the dark. **Round vacuolar inclusions were measured and classified according to the size range measured in microns (μm) that they fell into. Blue and red bars represent the AVI sizes in dark- and light-grown cells, respectively.

Comparison of the size of the vacuolar inclusions in the dark- and light-grown BMS^35S::R+35::C1 ^cells (Fig. 7) shows that the majority of the inclusions in the dark grown samples are in the size range of 0.1 μm – l μm, with a noticeable absence of larger ones. The modal range of the vacuolar inclusions in light grown cells was 2 μm – 3 μm, with some as large as 14 μm. This may reflect the fusion of smaller vacuolar inclusions to give rise to larger ones (see below). The control BMS cells, grown in the dark or light, did not show any evident sub-cellular morphological changes (not shown).

**Figure 8:**
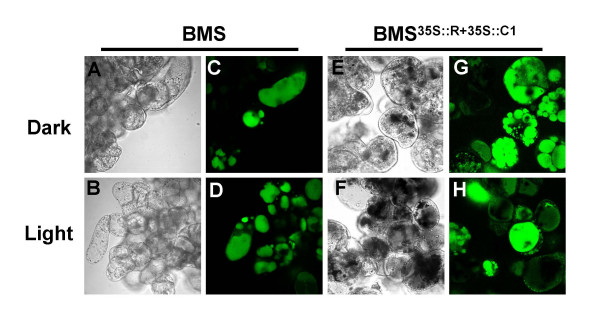
Morphology of vacuoles of dark and light grown BMS and BMS^35S::R+3ss::C1 ^cells loaded with BCECF-AM.

To further understand the changes induced by light in anthocyanin-accumulating cells, we performed laser scanning confocal microscopy of BCECF-AM loaded BMS^35S::R+35::C1 ^cells. Dark-grown cells showed the presence of multiple vacuoles (Fig. 8G), which, upon light treatment, appeared to coalesce to form fewer, much larger vacuoles (Fig. 8H). Light-grown cells display a decrease in fluorescence, likely because of quenching by the anthocyanins released from the vacuolar inclusions. Dark- or light-grown BMS cells showed no distinctive differences in visual fluorescence intensity or vacuolar morphology (Fig. 8 C, D), suggesting that the observed morphological alterations are either a consequence of the expression of the R and C1 regulators, of the accumulation of anthocyanins or of distinctive properties of the vacuoles in which anthocyanins accumulate.

Together, these results show that light-exposed BMS^35S::R+35::C1 ^cells have a significant reduction in the number of vacuoles with a associated increase in their size, a change in the number, shape and size of the AVIs and a release of anthocyanins from the AVIs into the vacuole.

### Light-induced vacuolar morphological alterations in anthocyanin-accumulating maize floral organs

**Figure 9:**
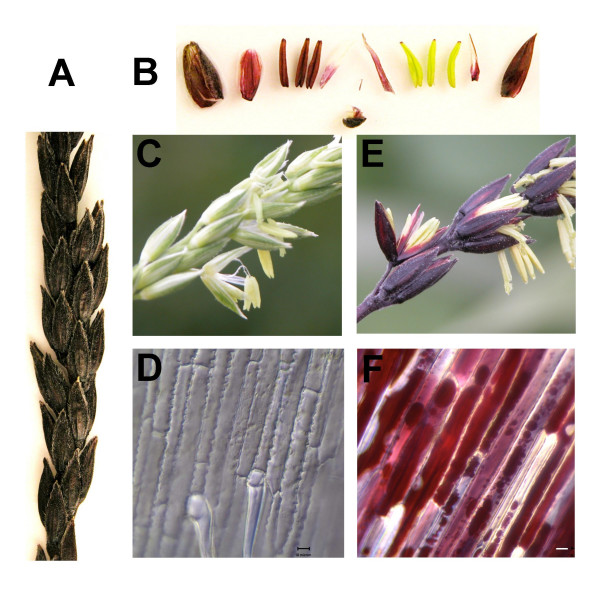
**Morphology of anthocyanin-accumulating cells in maize floral organs. **The maize male inflorescence, the tassel, is a panicle made of numerous spikes, each formed by numerous, paired spikelets. (A) Single spike with paired spikelets. The spikelet has outer and inner glumes (bracts of the florets) and each floret has a lemma, a palea, a highly reduced lodigule and 3 stamens (B) Spikelet dissection: the two florets with an outer glume, an inner glume; each floret with a lemma, palea, highly reduced lodigule and three stamens. (C) Digital macro images of open florets from a *C2-Idf *(chalcone synthase) mutant that accumulates no anthocyanins. (D) DIC light micrographs of the lemma from male flowers of *C2-Idf *plant (E). Digital macro images of open florets from a *B-I Pl *plant. (F) DIC light micrographs of the lemma from male flowers of a *B-I Pl *plant. The bar represents 10 μm

To establish whether the light-induced vacuolar alterations observed in BMS cells could be observed also *in planta*, we looked at the tassels of maize *B-I Pl *plants that accumulated large quantities of anthocyanins (Fig. 9). The inner, light-protected lemma and palea (Fig. 9B) were the choice of material to observe the light-induced alterations in vacuolar morphology. The epidermal cells of these appendages in a *C2-Idf *mutant lacking anthocyanins (Fig. 9C) have one to a few large, observable, colorless, central vacuoles (Fig. 9D). In contrast, depending on the physiological and developmental stage of the florets, the epidermal cells of the lemma or palea of the *B-I Pl *florets (Fig. 9B, E) were either already filled with anthocyanins, or were in the initial stages of accumulation (Fig 9F). These cells show a distinctive multi-vacuolar morphology, and the vacuoles were often heavily loaded with anthocyanins and AVI-like structures (Fig. 9F).

**Figure 10:**
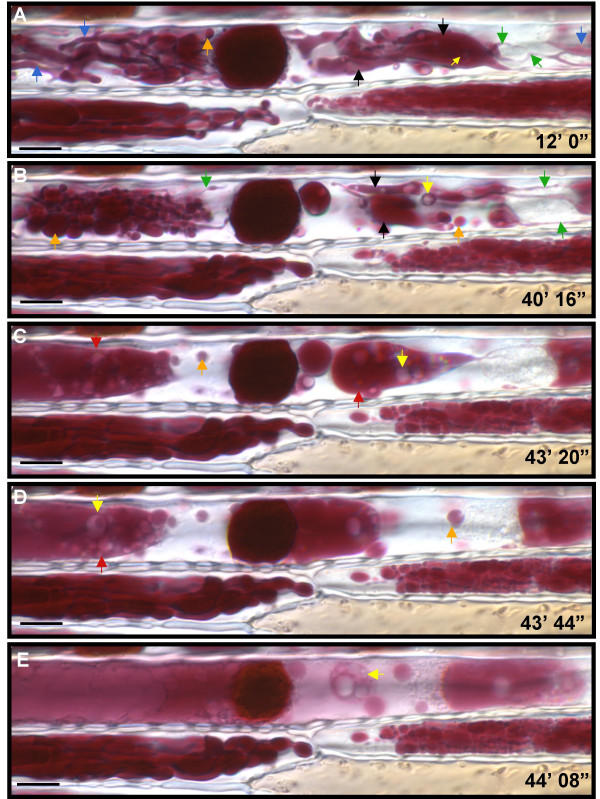
**Sub-cellular morphology of *BI Pl *maize floral cells accumulating anthocyanins. **DIC images of a maize lemma from B-I/B-Peru plant over-accumulating anthocyanins. The above are extracted images from a time-lapse series (See Additional file 1: Movie 1). The time points on the images indicate the period from time 0' i.e. when the sample was mounted onto the stage and exposed to the microscope light. (A) and (B) occur earlier in the series while (D), (C) and (E) are in rapid succession (24 seconds apart). The large central inclusion corresponds to a vacuolar inclusion containing anthocyanins measuring 15 μm in diameter. The green, blue and black arrows indicate, in that order, sequential stages in the conversion of thin tubular anthocyanin-filled structures to thick sheet-like structures. The orange arrows indicate the next step, which is the conversion into round structures. The red arrows indicate large fusion bodies resulting from the fusion of the swollen round structures. The yellow arrows point to clear spherical structures devoid of anthocyanins. The bar represents 10 μm.

Light from the microscope was sufficient to induce dramatic alterations in the morphology and distribution of the anthocyanin-containing vacuolar structures, as seen in the time-lapse images taken at four-second intervals (Fig. 10, see Additional file 1: Movie 1 for the original data used to perform this analysis). In these series, the initially thin, tubular anthocyanin-filled structures (average diameter of 0.6 μm, Fig. 10, green arrows) expand to a thickness of about 1.4 μm, dynamically filling the entire cell (Fig. 10A, blue arrows). These thick, finger-like projections became swollen, sheet-like structures (~3.3 μm in diameter, Fig 10, black arrows), to then become rounded (Fig 10A, B, C; orange arrows) and fuse with each other. These rounded/oval compartments measuring one 1–9 μm in diameter displayed, just like the tubular structures, dynamic morphological changes. Swollen "blebs" were observed moving along fine tubules and the ends of the thick tubules swelled up into round structures. Fusion events, once initiated, were very rapid, which resulted in the formation of large fusion bodies (Fig. 10C, D, E; red arrows) containing numerous clear (i.e., no anthocyanin-containing) structures (Fig 10C, D, E; yellow arrows). These clear inclusions were also observed initially in the sheet-like structures and were formed as the size of the tubules grew. The fusion bodies progressed rapidly to fill the entire cell, finally coalescing together resulting in the anthocyanin spread throughout the compartment (Fig 10E). The defined margins around the large central AVI-like structure (~15 μm across) become more diffuse with a lighter translucent red halo around the opaque, dark body (Fig. 10E).

In contrast to the BMS^35S::R+35S::C1 ^cells, in which anthocyanin production was uncoupled from the light-induced morphological alterations, the accumulation of anthocyanins in these *B-I Pl *cells is light induced [23]. Thus, the observed alterations in vacuolar morphology could be a consequence of the light-induced expression of the transcription factors, of the light-induced accumulation of anthocyanin, of light-induced alterations in vacuolar morphology observed in BMS^35S::R+35S::C1 ^cells, or a combination of them.

## Discussion

Anthocyanin pigments play many important eco-physiological roles in plants. While the biosynthesis and regulation of anthocyanins has been extensively described, little is known on how these pigments are sequestered in the vacuole and to what extent their modes of storage affect color. We describe here cytological changes of vacuoles and sub-vacuolar compartments containing anthocyanins in maize cells exposed to light.

Our studies in BMS^35S::R+35S::C1 ^cells show anthocyanins to accumulate even in total darkness, without any effect of light on the levels of anthocyanins nor on the amount of transcripts of the various flavonoid biosynthetic genes. From these results, we conclude that, when the R and C1 regulators are expressed, there is no need for additional light-induced factors to influence the control of the pathway. This provides strong support to previous findings suggesting that the flavonoid pathway is regulated by light at the level of transcription of the known R2R3 MYB and/or bHLH transcriptional activators and not at the level of the pathway structural genes [23,25,26]. Thus, the BMS^35S::R+35S::C1 ^cells provide a powerful tool to uncouple the effect of light on anthocyanin accumulation and pigmentation, something not feasible in most plant systems, where anthocyanins are induced by light.

The similar levels of anthocyanins in the BMS^35S::R+35S::C1 ^cells under dark or light conditions allowed us to uncover a second effect of light on pigmentation. Light-treated BMS^35S::R+35S::C1 ^cells were darker in color, when compared to identical cells grown in the dark (Fig. 2). Significant quantifiable reflectance differences are observed between BMS^35S::R+35S::C1 ^cells grown for six days in continuous light or dark (Table 1), changes that are not associated with a variation in the amount or type of anthocyanins present (Fig. 3). Microscopy studies established that extensive vacuolar morphological alterations (Fig. 6) correlate with the color darkening. BMS cells not expressing the anthocyanin regulators usually have one or a few vacuolar compartments (Fig. 5). In contrast, constitutive expression of R and C1 in these cells results in a remarkable increase in vacuolar number. It remains to be established whether the accumulation of flavonoids/anthocyanins, the expression of the regulators, or both are necessary and sufficient to trigger the biogenesis of new vacuolar compartments. Within the vacuoles, anthocyanins accumulate in maize cells in red spherical bodies that resemble vacuolar anthocyanoplasts [27]. Although we have not yet unequivocally established whether the anthocyanin inclusions present in BMS^35S::R+35S::C1 ^cells are membrane-bound or not, they have very similar characteristics to the recently-described yellow auto-fluorescent bodies (YFB) present in the vacuoles of BMS cells, induced by the expression of the P1 regulator of 3-deoxy flavonoid biosynthesis [30].

The organization of the anthocyanin inclusions present in BMS^35S::R+35S::C1 ^cells undergo dramatic modifications in the presence of light. These alterations include a reduction in their numbers and an enlargement of their size (Fig. 6). In addition, light-treated vacuoles often showed a spreading of the anthocyanin pigment within the vacuolar lumen, which may be a result of release from the AVI-like structures. The morphological changes observed in the vacuoles of BMS^35S::R+35S::C1 ^cells are not a "curiosity" of cells in culture. We uncovered similar light-induced alterations in the vacuolar structure of maize floral tissues accumulating high levels of anthocyanins (Fig. 8, 9). Time-lapse experiments (see Additional file 1: Movie 1) illustrate the formation and fusions of tubular and globular anthocyanin-filled structures that ultimately coalesce to give one or a few large central vacuoles characteristic of pigmented maize cells. These tubular-vesicular structures, some of which are filled with clear vesicles, are reminiscent of observations of the tubular provacuoles found in vacuolating *Euphorbia *root cells [8,31]. The anatomical identity of these structures remains to be established, but similar to what was reported for the *Euphorbia *root cells, the ontogenesis of larger anthocyanin-containing vacuoles from smaller ones is reminiscent of vacuolar fusion and/or autophagy.

It is possible that these light-induced morphological alterations in the anthocyanin-containing structures are directly associated, if not responsible, for the observed color changes. However, alterations in vacuolar pH or the association of anthocyanins with co-pigments can also result in changes in the hue of anthocyanin pigmentation [13,14]. To investigate whether light induces a change in the vacuolar pH of BMS cells, we utilized the BCECF-AM fluorescent dye, for which the ratio of fluorescence emission at the dual excitation wavelengths of 490 nm/440 nm can be calibrated to an *in vivo *generated pH curve [32]. Using this method, we established that the vacuoles of dark and light grown BMS cells were acidic at pH 5.8 and showed no significant pH differences (see Additional file 2: Fig. 1). Similarly, pH measurement of crude homogenates of BMS or BMS^35S::R+35S::C1 ^cells in water did not yield significant pH value differences between each other or between light- and dark-grown cells (data not shown). Although unlikely, based on the absence of a shift of the λ_max _of the pigments (Fig. 3 A, B), we also investigated whether light participated in the induction of phytochemicals that could serve as co-pigments, and hence contribute to the color change. Reverse phase HPLC analyses exhibited no significant differences in the peak profiles of phenolic compounds in the dark and light grown BMS and BMS^35S::R+35S::C1 ^cells (not shown).

Although these results do not rule out the possibility of a local and/or transient light-induced pH change or the induction of a minor co-pigment responsible for the color shift, one possible explanation is that the alterations in vacuolar morphology are the cause for the light induced darkening phenomenon of the anthocyanin containing cells. Similar light-induced effects were previously described for the anthocyanoplasts of red cabbage, an observation that was attributed to an increased accumulation of anthocyanins enhanced by light [9]. Our results, however, offer the alternative explanation that light itself can affect the packaging and distribution of anthocyanins within the vacuole, independently of variations in the levels of anthocyanins. Similarly, flowers of the "Rhapsody in Blue" rose cultivar show a change in color induced by age, from red-purple to bluish-purple, and this variation was associated with a progressive accumulation of anthocyanins into AVI-like structures [29]. *Lisianthus *flowers also show a correlation between the packaging of anthocyanins into AVIs, the presence of "blackish-purple" pigmentation at the base of the petal, and the reduction or absence of AVIs in the outer zones, associated with a lighter purple color of this region [11]. It is thus possible that the observed alteration in the hue of light-grown BMS^35S::R+35S::C1 ^cells reflects a much more general mechanism of light on the packaging of anthocyanins within the vacuole, and hence on pigmentation. If so, the BMS^35S::R+35S::C1 ^cells provide a convenient system for the dissection of the mechanisms of this process because of the molecular and cellular tools available.

## Conclusion

The results presented here provide evidence that light affects anthocyanin pigmentation by mechanisms beyond the transcriptional regulation of genes encoding pathway enzymes. In maize floral organs and cultured cells, light induces dramatic morphological alterations in the packing of anthocyanins and distribution of vacuolar and sub-vacuolar compartments. Similar phenomena have been observed before, but the difficulties associated in uncoupling anthocyanin production with morphological alterations in their packaging prevented to draw conclusive cause-consequence relationships.

## Methods

### Growth, maintenance and treatment of BMS cells

BMS cells were maintained in conditions previously described [33]. In brief, BMS cells were sub-cultured every seven days in liquid MS media supplemented with 2,4,D (0.5 g/L), 3% sucrose (BMS media) on a rotatory shaker (150 rpm) in the dark at 25 ± 2°C. For dark and light treatments, cells from suspension cultures were plated on filter paper overlaid on BMS solid media containing 0.3% phytagel, and were allowed to establish for 20 days in darkness at 25 ± 2°C. Plates were shifted to total darkness (covered with alumnum foil) or light at 50 ± 5 μmol.m^-2 ^_._s^-1 ^(Cool white. 215W, F96T12/CW/VHO, Sylvania, Canada).

### Transient expression experiments

BMS suspension cells (3 g of cells in 25 ml of BMS media) were treated overnight with 1.7% PEG in BMS media. One ml of cells was overlaid on pre-soaked filter papers in Petri plates. Ten micrograms of 35S::R+35S::C1 plasmid (pPHP687 in [27]) was coated onto gold microprojectiles according to the manufacturers recommendations (Bio-Rad Laboratories, Inc., USA). Coated gold particles were bombarded into PEG-treated BMS cells using a Biolistic PDS-1000/He particle gun (Bio-Rad Laboratories, Inc. USA) at 1,100 psi. The plates were kept in the dark (covered with foil) or exposed to light for a period of 24 hr, after which cells were analyzed microscopically.

### Reflectance analysis

*In vivo *reflectance measures were taken with a Minolta CR-300 reflectometer/colorimeter (Minolta, Japan). The color was represented as CIEL*a*b* values (for the CIE D65/10° illuminant/observer condition). The L* value represents the lightness level, ranging from 100 (white) to 0 (black), the a* (+a red; -a green) and b* (+b yellow; -b blue). The instrument was normalized to standard white tile provided with the instrument before performing analysis on cells grown on solid BMS media in Petri plates.

### Extraction and analysis of anthocyanin pigments

BMS^35S::R+35S::C1^ or control BMS cells after a light or dark treatment were lyophilized for 36 hrs. Anthocyanins and other phenolics were extracted in 50% methanol overnight at a ratio of 50 μg of dry tissue per μl of methanol. Methanol extracts were diluted in 1% HCL in 50% methanol and absorption spectra were collected between 400 to 700 nm with 5 nm intervals at 0.5 s with a Cary 50 UV-VIS spectrophotometer (Varian, Inc. USA). Graphs were generated using the Cary WinUV software. Anthocyanins were measured spectrophotometrically at 530 nm. For the generation of the anthocyanidins from the corresponding anthocyanins, methanol extracts were hydrolyzed by the addition of an equal volume of 2 M HCL (37% v/v) and heated in a boiling water bath for 20 minutes. Hydrolyzed samples were extracted with isoamyl alcohol. Chromatographic separation of the anthocyanidins was performed by thin layer chromatography (TLC) on cellulose plates (5730/7, EM Science, Germany) with HCL/formic acid/H_2_O, 3:30:10, v/v as the mobile phase. Twenty μL of methanolic extracts of non-hydrolyzed and hydrolyzed samples were injected into a Waters Alliance^® ^2695 Separations module (Waters Corporation, Milford, MA) in conditions as described [34]. The HPLC profiles were obtained at 280 nm using the Waters 2996 Photodiode Array Detector and analyzed with the Empower software (Waters Corporation, Milford, MA).

### Extraction and analysis of RNA

Dark and light grown BMS cells were homogenized in liquid nitrogen and total RNA was extracted using the TRIzol reagent following the manufactures recommendations (Invitrogen, Life Technologies, USA). For Northern analyses, 25 μg of total RNA was separated on a formaldehyde-containing 1% agarose gel and blotted onto a nitrocellulose membrane (Bio-Rad Laboratories, Inc., USA). The blot was hybridized with cDNA probes corresponding to *c2 *[35], *f3h *[36] and *a1 *[37]. Ubiquitin [38] was used as a normalization control. Comparison of the hybridization signals was performed on a BioRad phosphorimager (BioRad Laboratories, Inc., USA) and ratios of the dark and light grown callus hybridization signal to the ubiquitin normalization control were compared.

### Plant material

Maize kernels for *C2-Idf *the genetic stock 418D C2-Idf1 (Active-1); *A1 A2 C1 R1 *and *BI Pl- *219I *B1-I; A1 A2 C1 C2 Pll-Rhoades rl-r, 219J B1-I; A1 A2 C1 C2 Pl1-Rhoades r1-g *were obtained from the Maize Coop . The kernels were planted in the field in the summer and just before anthesis, male tassels were collected for observation of vacuolar structure of the lemma or the palea.

### Microscopy analysis and vacuolar staining

Digital images of the maize floral whorls and callus cells were captured with a Nikon COOLPIX 5700 camera. Macroscopic images of the transient experiments were visualized with an Olympus SZH10 Research Stereo microscope (Olympus, Japan) and images were captured with a Olympus DP10 digital camera. Light and dark grown BMS cells were examined under a Nikon Eclipse E600 microscope. Differential interference contrast (DIC) pictures were taken with a SPOT, RT-Slider digital camera and analyzed using the SPOT imaging software (Diagnostic Instruments, Inc., USA). DIC time lapse images were taken every 4 seconds for 2 hours and were converted into a movie using the SPOT imaging software. For vacuolar staining, transformed and control BMS cells were incubated with 10 μM BCECF-AM (Molecular Probes, USA) in BMS media for 40 minutes at room temperature. Cells were spun down, washed twice and re-suspended in BMS media. Laser scanning confocal microscopy with a PCM 2000/Nikon Eclipse600 system (Nikon Bioscience Confocal Systems, NY) was used to capture digitized images of the BCECF stained cells using the Nikon Plan Fluor 40X/0.75 air objective (1 pixel = 0.3 μm) as described [39]. The 488 nm excitation wavelength of the argon laser was used in conjunction with a 515/30 nm bandpass emission filter (EM515/30HQ). Images were captured using the SIMPLEPCI software (Compix Imaging Systems, PA) and assembled using Adobe PHOTOSHOP (Adobe Systems, Mountain View, CA).

### pH measurement

BMS cells (6 ml) were grown in 35 mm Petri plates at a concentration of 0.lg/ml (fresh weight/vol.) for 6 days under light (50 μmol.m^-2^.s^-1^) and dark (foil covered) at 100 rpm. Cells were filtered, weighed and resuspended at a concentration of 0.1 g/ml. One ml of cells were loaded with 10 μM BCECF-AM as described above. Hundred μl of loaded and washed cells were pipetted into a 96 well microtitre plate. An *in situ *calibration curve was generated separately for each of the replicates for the dark and light grown BMS cells. 100 μl of 0.1 M of various pH buffers from a range of 5.0 to 7.0 with 0.005% digitonin [40] was added to 100 μl of cells, and incubated for 10 min. Fluorescence emission was measured at 535 nm with excitation at 440 nm and 490 nm using the FLEX station™ and data analysis program SOFTmax PRO 4.3 (Molecular Devices, CA). The emission ratio at 490/440 was used for calculation of the pH, where irregularities due to unequal loading are eliminated. These measurements were carried out in triplicate.

## Authors' contributions

NGI carried out all the molecular and cellular experiments described. EG conceived the project and participated in the design and coordination of the study.

## Supplementary Material

Additional File 1 Time-lapse DIC images of a maize lemma from *B-I Pl *plant over-accumulating anthocyanins. Images were taken every four seconds. The time-lapse series was converted into a movie at one frame per second, therefore a speedup of 4X real time. The last four images represented in Fig. 10(C, D and E) are from this time-lapse series. Note the clear round inclusions in the tubular structures and the large vacuoles at the end. Refer to the results section for a detailed description. **Viewing Instructions: **The movie can be visualized using either Quicktime player or Windows Media Player. The plug-ins can be downloaded from  (Quicktime Player) or  Player). The movie size is ~2 MB, therefore it is more efficient to view by downloading the movie to your hard drive. Click here for file

Additional File 2 Figure 1. ***In situ *measurement of vacuolar pH using BCECF-AM. **Equal amounts (0.01 g/100 μl fresh wt) of BCECF AM loaded cells were placed in microtiter plates, and the emission measured at 535 nm, 440 nm and 490 nm excitation wavelengths (A). The 490/440 ratio was calculated (B). An *in situ *calibration curve was generated separately for each of the dark and light samples with various pH buffers with 0.005% digitonin (C). Vacuoles of both dark and light grown BMS cells were acidic at pH 5.8 and showed no significant pH differences (D). Click here for file
