# Supplementary figures and images for: Light-induced morphological alteration in anthocyanin-accumulating vacuoles of maize cells
Source: BMC Plant Biol. 2005 May 20;5:7. doi: 10.1186/1471-2229-5-7 (PMC1177971; doi:10.1186/1471-2229-5-7)

## Slide 1
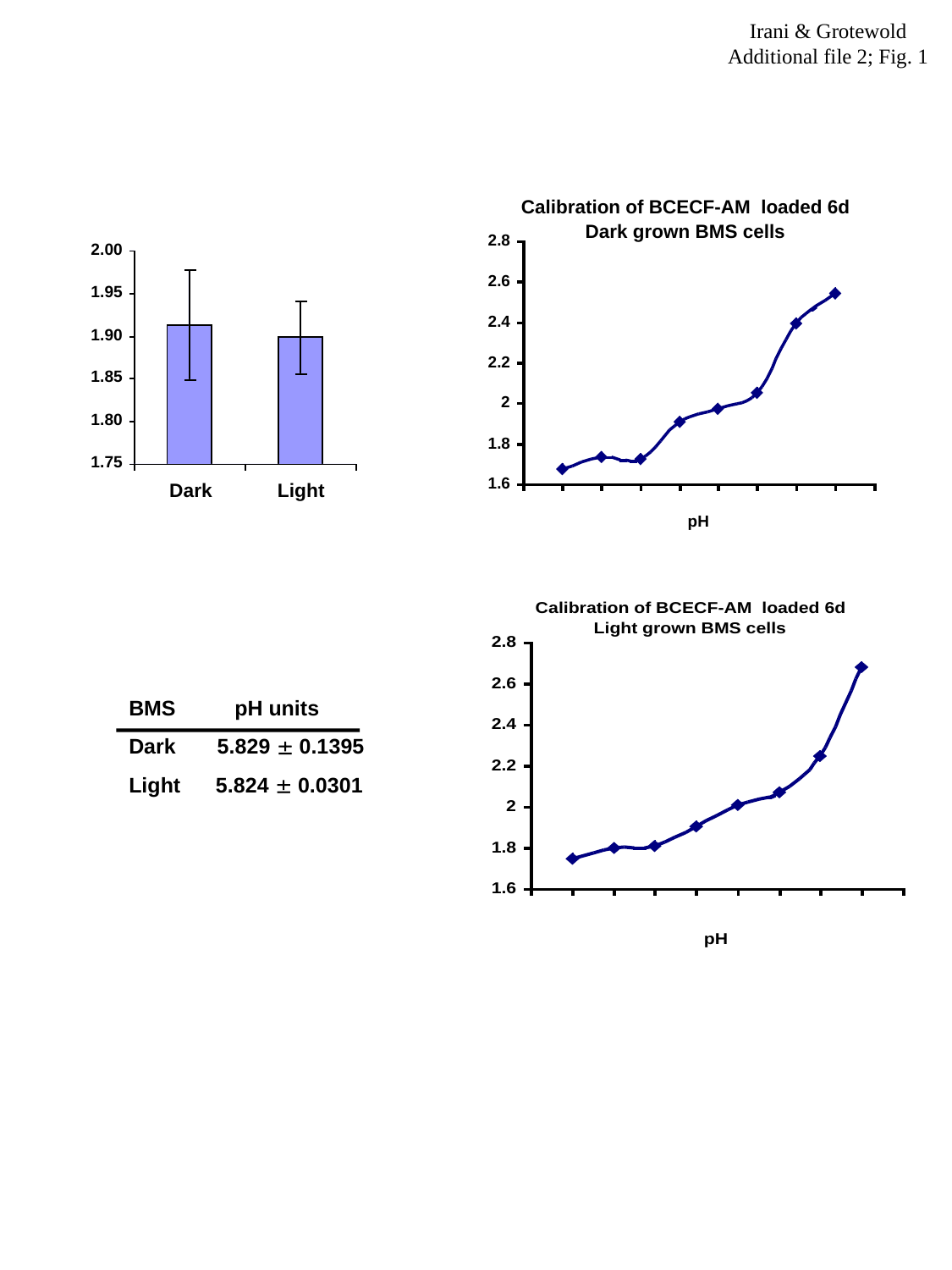

Irani & Grotewold
Additional file 2; Fig. 1
BMS pH units
Dark 5.829 ± 0.1395
Light 5.824 ± 0.0301

Supplement: Additional File 2 — Figure 1. In situ measurement of vacuolar pH using BCECF-AM. Equal amounts (0.01 g/100 μl fresh wt) of BCECF AM loaded cells were placed in microtiter plates, and the emission measured at 535 nm, 440 nm and 490 nm excitation wavelengths (A). The 490/440 ratio was calculated (B). An in situ calibration curve was generated separately for each of the dark and light samples with various pH buffers with 0.005% digitonin (C). Vacuoles of both dark and light grown BMS cells were acidic at pH 5.8 and showed no significant pH differences (D). [file 1471-2229-5-7-S2.ppt]
